# Bilateral Tibial Tuberosity Periosteal Sleeve Fracture in an Adolescent: A Case Report

**DOI:** 10.5704/MOJ.2311.013

**Published:** 2023-11

**Authors:** M Luqman, AF Rasid, K Jamil, AH Abd-Rashid

**Affiliations:** Department of Orthopaedics and Traumatology, Universiti Kebangsaan Malaysia, Kuala Lumpur, Malaysia

**Keywords:** tibial tuberosity, avulsion fracture, periosteal sleeve, adolescent

## Abstract

Tibial tuberosity avulsion fracture is a rare injury, and bilateral occurrence is more uncommon. Periosteal sleeve fracture is a unique fracture pattern which was first described in the lower pole of patella in children. We are reporting a rare case of bilateral tibial tuberosity sleeve fracture in a teenage boy which occurred while sprinting. The patient underwent open reduction, pull through suture fixation of the bilateral tibial tuberosity and screw fixation of left tibial tuberosity. Post-operative rehabilitation included gradual increment of range of motion with hinged brace and quadriceps muscle strengthening. Close follow-up was done to monitor the progression of his recovery. At six months follow-up, the patient recovered well. Both knees had full range of motion with an intact extensor mechanism.

## Introduction

Tibial tuberosity avulsion fractures are mostly seen in adolescent age group. It accounts for 0.4% to 2.7% of pediatric fractures and <1% of physeal injuries^1^. Partial sleeve fractures represent a fracture pattern characterized by the avulsion of cartilage or periosteum with associated small subchondral osseous fragment. This type of fracture is similar to Modified Ogden Type V, either Va (non-displaced) or Vb (displaced) fracture^2^, where the common mechanism of injury involves jumping or sprinting during sports activity. This case report describes the acute management and rehabilitation process in an adolescent patient who suffers this rare injury.

## Case Report

A 13-year-old boy complained of bilateral knee pain whilst playing indoor soccer. During a sprint, he heard a sudden cracking-like sound coming from his knees. He was able to weight bear initially, but unable to as the pain worsened. Physical examination revealed bruises around the infrapatellar region associated with minimal effusion in the suprapatellar recess. The extensor mechanism of both knees was also compromised.

**Fig 1:**
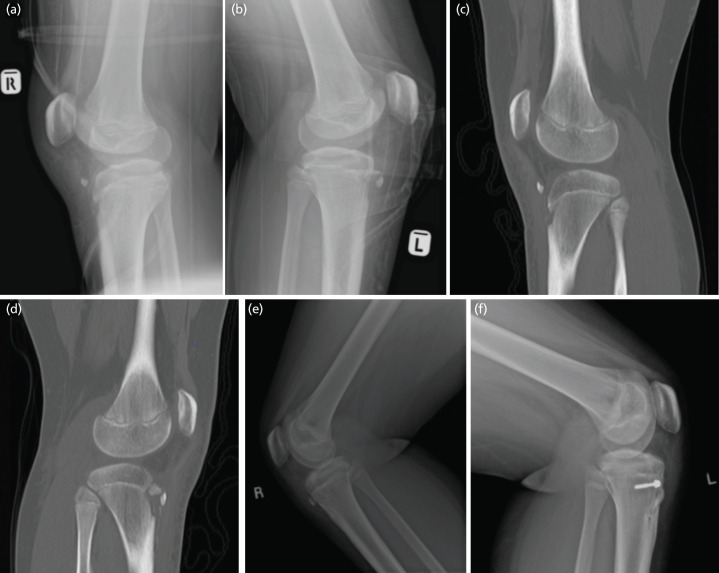
(a, b) Lateral view plain radiograph of right and left knee showing the displacement of the tibial tuberosity avulsion fracture. (c) CT sagittal view of right knee showing avulsed fragment at right knee measuring 0.5 x 1.4 x 0.8cm with approximately 2.4cm displacement from the tibial tuberosity. (d) CT sagittal view of left knee showing two fragments were seen at left knee measuring 1.0 x 0.4 x 1.5cm and 1.1 x 2.1 x 1.5cm, respectively, which were 1.2cm displaced. No intraarticular extension seen on both knees. (e, f) Lateral view plain radiograph at six months post operation showing reduced tibial tuberosity fragment of bilateral knees with in-situ screw at left tibial tuberosity.

Knee radiographs revealed bony fragments at the anterior aspect of both knee joints with associated tibial tuberosity irregularities (Fig. 1a and b). A computed tomography (CT) scan was done to assess the size of the avulsed fragments for operative planning. The scan showed an avulsed fragment at the right knee measuring 0.5 × 1.4 × 0.8cm with an approximately 2.4cm displacement from the tibial tuberosity (Fig. 1c). Two fragments were observed at the left knee measuring 1.0 × 0.4 × 1.5cm and 1.1 × 2.1 × 1.5cm, both of which were displaced by 1.2cm (Fig. 1d). No intra-articular extension was seen.

**Fig 2:**
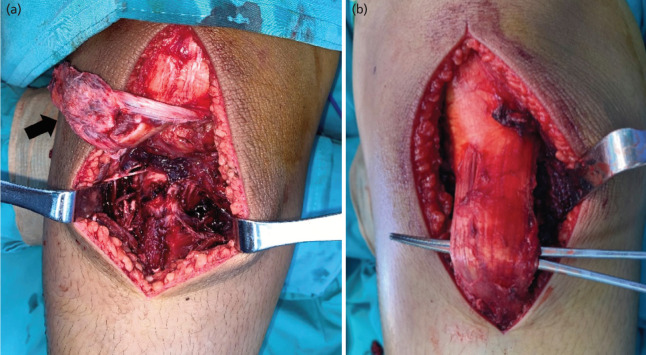
(a) Intra-operative picture of right knee showing a complete avulsion of the patella tendon with a large periosteal flap (black arrow). (b) Intra-operative picture of left knee showing patellar tendon was still attached at the tibial tuberosity but was stretched and became elongated.

Open reduction was planned for both knees. Intra-operatively, the right knee revealed a complete avulsion of the patella tendon with a large periosteal flap (Fig. 2a). However, the bony fragment was too small; thus, the patella tendon was repaired at the tibial tuberosity with pull-through suture method using non-absorbable polybraided suture (Ethibond) size 5. In the left knee, the patellar tendon was still attached to the tibial tuberosity, but it was stretched and became elongated (Fig. 2b). Two avulsed fragments were found, where one was large enough for screw fixation. Subsequently, a similar pull-through suture technique was performed to augment the patella tendon to the tibial tuberosity. Bilateral cylinder slab was used to maintain the knee joint in full extension.

**Fig 3:**
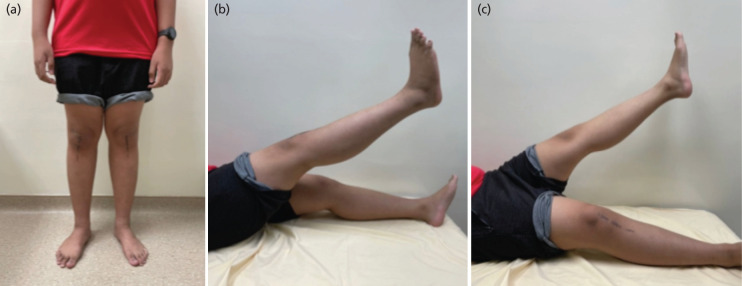
(a) Clinical pictures at six months post-operation showing that patient can stand up without any aid. (b, c) The patient able to extend his bilateral knees showing his intact extensor mechanism.

Post-operatively, the slabs were replaced with knee braces locked at 5° flexion. The patient was discharged on a recliner wheelchair with lower limb extension. During follow-up, gradual increment of range of motion of the knees was allowed. At two weeks post-op, he was allowed 0°–30° range of motion. Physiotherapy for muscle strengthening focusing on quadriceps strengthening was started at this juncture. After six weeks, the patient was permitted 90° knee flexion and allowed for full range of motion without the knee brace. After three months, full weight-bearing commenced aided with hinged brace. At six months follow-up, the patient was able to ambulate well without aid. His bilateral knee extensor mechanism and range of motion were fully restored (Fig. 3a – c). Lateral view plain radiograph at six months post-operation demonstrated reduced tibial tuberosity fragment of bilateral knees with in situ screw at the left tibial tuberosity (Fig. 1e and f).

## Discussion

Davidson and Letts were the first to highlight sleeve fractures occurring in the tibial tuberosity of three adolescent boys. All of them had good outcome following open reduction, multiple screw fixations and periosteal sutures. In this case, the pull-through sutures were the main fixation method, as only one bone fragment was amenable using a screw. Tibial tuberosity sleeve fracture should always be managed with an open reduction, as there is no bone fragment large enough to be reduced by closed reduction method^3^.

It is likely that sleeve fractures share the same mechanism and risk factors with avulsion fractures of the tibial tuberosity. There are few proposed risk factors for adolescents to obtain this injury. Those who are active in sports, obese, or have an underlying Osgood–Schlatter disease are more prone to suffer from this injury^4^. The patient in this study is active in sports and has a BMI of 29. However, he had no symptoms to suggest an underlying Osgood–Schlatter disease. Sudden burst of movement while sprinting may cause forceful contraction of the quadriceps, which leads to the fracture. The different severity of fracture suggests that the patient may have injured the right knee first, but the momentum from running brought about the subsequent left knee injury to a lesser degree as he decelerates. Being overweight increases the risk for bilateral injuries.

Post-operatively, cautions were taken in preventing failure of the fixation. The knee joint was protected using a hinged brace for 12 weeks. Patient was allowed partial weight-bearing at six weeks and full weight-bearing at 12 weeks. Gradual knee flexion was allowed with an increment of 30°, and he was allowed full range of motion of the knee at 12 weeks post-operation.

Bilateral tibial avulsion fractures carry a higher risk of complications compared to unilateral injuries. Kushare *et al* reported complications in 32.4% of bilateral cases, with hardware removal and wound dehiscence being the most common. Patients with bilateral injuries also had longer recovery durations and periods of immobilization, requiring a wheelchair for ambulation, resulting in a delayed return to school^5^. However, the patient in this study did not experience any complications during the six-month follow-up, had already resumed schooling, and could perform light jogging.

The authors would like to highlight that this rare bilateral injury may occur without significant trauma, e.g., while running. In this case report, the protocol of open reduction, pull-through sutures, and screw fixation, followed by gradual increment of knee range and physiotherapy have led to a good early clinical outcome.
